# The *Candida albicans* Cdk8-dependent phosphoproteome reveals repression of hyphal growth through a Flo8-dependent pathway

**DOI:** 10.1371/journal.pgen.1009622

**Published:** 2022-01-04

**Authors:** Jeffrey M. Hollomon, Zhongle Liu, Scott F. Rusin, Nicole P. Jenkins, Allia K. Smith, Katja Koeppen, Arminja N. Kettenbach, Lawrence C. Myers, Deborah A. Hogan

**Affiliations:** 1 Department of Microbiology and Immunology, Geisel School of Medicine at Dartmouth, Hanover, New Hampshire, United States of America; 2 Department of Biochemistry and Cell Biology, Geisel School of Medicine at Dartmouth, Hanover, New Hampshire, United States of America; 3 Norris Cotton Cancer Center, Norris Cotton Cancer Center, Dartmouth-Hitchcock Medical Center at Dartmouth, Lebanon, New Hampshire, United States of America; 4 Department of Medical Education, Geisel School of Medicine at Dartmouth, Hanover, New Hampshire, United States of America; University of Texas Health Science Center at San Antonio, UNITED STATES

## Abstract

Ssn3, also known as Cdk8, is a member of the four protein Cdk8 submodule within the multi-subunit Mediator complex involved in the co-regulation of transcription. In *Candida albicans*, the loss of Ssn3 kinase activity affects multiple phenotypes including cellular morphology, metabolism, nutrient acquisition, immune cell interactions, and drug resistance. In these studies, we generated a strain in which Ssn3 was replaced with a functional variant of Ssn3 that can be rapidly and selectively inhibited by the ATP analog 3-MB-PP1. Consistent with *ssn3* null mutant and kinase dead phenotypes, inhibition of Ssn3 kinase activity promoted hypha formation. Furthermore, the increased expression of hypha-specific genes was the strongest transcriptional signal upon inhibition of Ssn3 in transcriptomics analyses. Rapid inactivation of Ssn3 was used for phosphoproteomic studies performed to identify Ssn3 kinase substrates associated with filamentation potential. Both previously validated and novel Ssn3 targets were identified. Protein phosphorylation sites that were reduced specifically upon Ssn3 inhibition included two sites in Flo8 which is a transcription factor known to positively regulate *C*. *albicans* morphology. Mutation of the two Flo8 phosphosites (threonine 589 and serine 620) was sufficient to increase Flo8-HA levels and Flo8 dependent transcriptional and morphological changes, suggesting that Ssn3 kinase activity negatively regulates Flo8.Under embedded conditions, when *ssn3*Δ/Δ and *efg1*Δ/Δ mutants were hyperfilamentous, *FLO8* was essential for hypha formation. Previous work has also shown that loss of Ssn3 activity leads to increased alkalinization of medium with amino acids. Here, we show that the *ssn3*Δ/Δ medium alkalinization phenotype, which is dependent on *STP2*, a transcription factor involved in amino acid utilization, also requires *FLO8 and EFG1*. Together, these data show that Ssn3 activity can modulate Flo8 and its direct and indirect interactions in different ways, and underscores the potential importance of considering Ssn3 function in the control of transcription factor activities.

## Author summary

In *Candida albicans*, Ssn3 kinase activity co-regulates the transcription of numerous genes involved in hyphal growth, metabolism and nutrient acquisition, immune cell interactions, and drug resistance. Using a strain in which Ssn3 could be rapidly and selectively inhibited, we identified both known and novel Ssn3 targets. We identified two phosphosites in Flo8, a regulator of morphology and virulence, that were shown to negatively regulate Flo8 levels and activity. The data and tools presented here will enable a better understanding of how Ssn3 impacts transcriptional and post-transcriptional regulation in order to coordinate processes during physiological and morphological transitions as well as during steady state growth.

## Introduction

One of the important roles of the Mediator transcriptional co-regulatory complex is to link the activity of promoter-bound transcriptional factors to the basal transcription machinery. The Cdk8 module of Mediator plays important roles in modulating the activity of Mediator itself as well as the activity of transcription factors, among other proteins. In *Candida albicans*, like in other eukaryotes, the Cdk8 module consists of four subunits: the catalytic subunit Ssn3 (Cdk8), Ssn8 (CycC), Med12 (Srb8), and Med13 (Srb9). Ssn3, the catalytic component of the Cdk8 module, is a cyclin-dependent like kinase and its activity depends on the cyclin-like protein Ssn8. Across eukaryotic species, the Cdk8 kinase has been shown to be particularly important for regulation during metabolism and morphology [1,2]. In mammals, for example, glycolysis, lipogenesis and immune responses are influenced by Cdk8 phosphorylation of specific regulators [3–5]. In *S*. *cerevisiae*, Ssn3 (Cdk8) has been well-studied for its roles in metabolism [6,7] and its negative regulation of pseudohyphal growth through its direct effects on Ste12 [8] and Phd1 [9,10]. The Cdk8 module is of particular interest for its role in transitions between growth conditions and during development when cells need to rapidly make coordinated changes to the activities of certain transcription factors at specific promoters as well as for its role in maintaining proper levels of constitutive promoter activation [11].

In *C*. *albicans*, null mutations in either *SSN3* or *SSN8* result in common, pleiotropic phenotypes. *SSN3* null mutants had increased induction of genes involved in drug resistance [12] and increased resistance to bacterially-produced metabolic inhibitors [13]. The *ssn3*Δ/Δ leads to a hyperwrinkled colony morphology, increased respiratory metabolism and amino acid utilization [13], and an increased fraction of Ras1 in its active GTP-bound state [14]. Mutation of *SSN3* was found to unmask an alternative filamentation pathway in macrophages [15]. Recent work by Lu and colleagues [16] found that changes in Ssn3 activity in response to CO_2_, mediated by the Ptc2 phosphatase, led to decreased Ssn3 phosphorylation and decreased inhibition of Ume6, a positive regulator of hyphal growth.

Here, we utilize analog-sensitive variants of *C*. *albicans* Ssn3, as has been performed in *S*. *cerevisiae* and in human cells, [3,17], to study the immediate effects of inhibition of Ssn3 kinase activity in *C*. *albicans*. Under conditions that do not promote hyphal growth in wild-type strains, Ssn3 inhibition led to the formation of hyphae. We used these conditions to elucidate the *C*. *albicans* Ssn3 regulon as it relates to the control of morphology using phosphoproteomic and transcriptomic analysis of cells shortly after Ssn3 inhibition. Flo8, a transcription factor that positively regulates hyphal growth [18,19], was identified in the phosphoproteomics analysis as a candidate for Ssn3 regulation, and transcriptomics data showed alterations in hypha-specific genes known to be regulated by Flo8. Deletion of Ssn3-phosphosites in Flo8 was sufficient to affect protein levels and morphology. Additional assays suggest that Flo8 plays major roles in Ssn3-regulated control of metabolism and that *ssn3*Δ/Δ phenotypes also require Efg1. The data in this manuscript indicate the spectrum of proteins that are altered, directly or indirectly, by Ssn3 kinase activity as cells respond to changing environments. While these studies focus on Ssn3 interactions with Flo8, our data show that other transcription factors, including but not limited to Efg1, Eed1, and Mrr1, are also likely Ssn3 targets. Thus, the tools presented here may aid in the study of diverse proteins involved in morphological transitions, metabolism, virulence and drug resistance.

## Results

### 3-MB-PP1 inhibits analog-sensitive Ssn3

To investigate the direct targets of the *C*. *albicans* Ssn3 kinase, we sought to develop a strain in which Ssn3 kinase activity could be rapidly inhibited using approaches that have been successfully applied in *S*. *cerevisiae* [17]. Based on the alignment of the *C*. *albicans* and S. *cerevisiae* Ssn3 orthologs, we predicted a phenylalanine to glycine substitution at position 257 would yield a variant that retained the functions of the wild-type kinase, while being able to be specifically inhibited by the ATP analog 3-MB-PP1 [20].The Ssn3^F257G^ was constructed and is henceforth referred to as the analog-sensitive variant, Ssn3^AS^ (see **S1 Table** for summary of strains).

**Fig 1:**
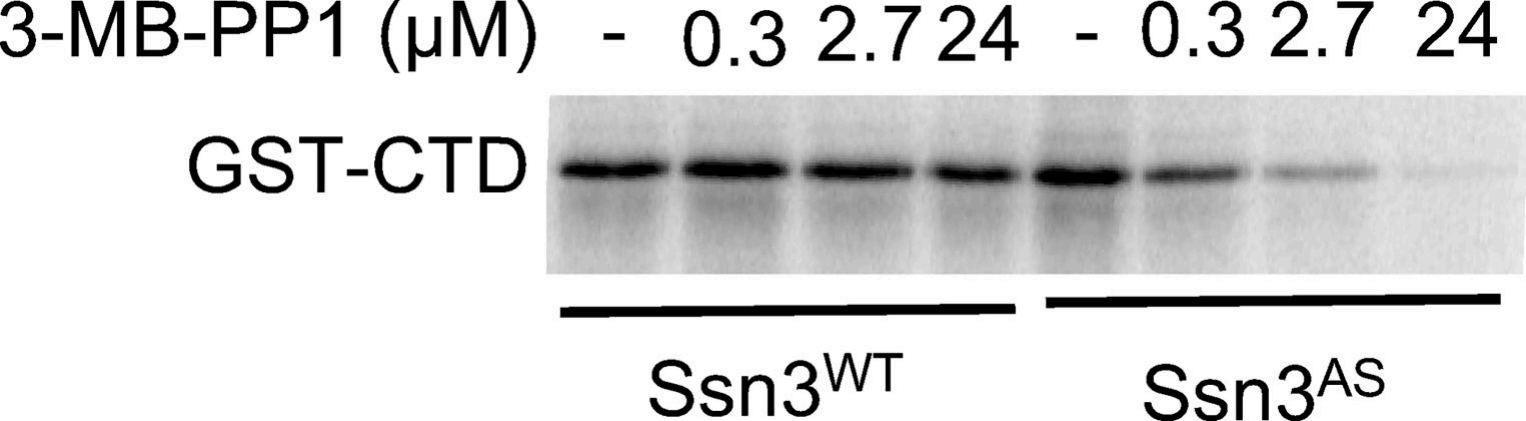
3-MB-PP1 inhibits the activity of analog-sensitive Ssn3^AS^, but not Ssn3^WT^*in vitro*. *In vitro* kinase reactions contained purified Mediator from a strain with Ssn3^WT^ or Ssn3^AS^, ^32^P-ATP, purified GST-tagged RNA Pol II C-terminal domain (CTD) and the indicated concentrations of 3-MB-PP1 inhibitor. Reactions were analyzed by SDS-PAGE and visualized by phosphorimaging.

We first assessed the inhibition of Ssn3^AS^ by 3-MB-PP1 with an *in vitro* kinase assay using purified Mediator complex containing the Cdk8 module. Ssn3^WT^ or Ssn3^AS^ were expressed in a background that contained a His-FLAG-tagged derivative of Ssn8 for purification. The activity of Ssn3^WT^ or Ssn3^AS^ was assessed using ^32^P *in vitro* kinase assays [21] in which phosphorylation of recombinantly produced C-terminal domain (CTD) of RNA Pol II, an Ssn3 substrate, was monitored. The Ssn3^WT^ and Ssn3^AS^ kinases had equal CTD phosphorylation activity in the absence of inhibitor (**Fig 1**). The Ssn3^AS^ kinase activity was inhibited by 3-MB-PP1 in a dose-responsive fashion, while addition of this compound had no effect on the Ssn3^WT^ kinase activity (**Fig 1**). A concentration of 2.7 μM 3-MB-PP1 inhibited ~85% of the Ssn3^AS^ activity and 24 μM virtually eliminated the activity.

**Fig 2:**
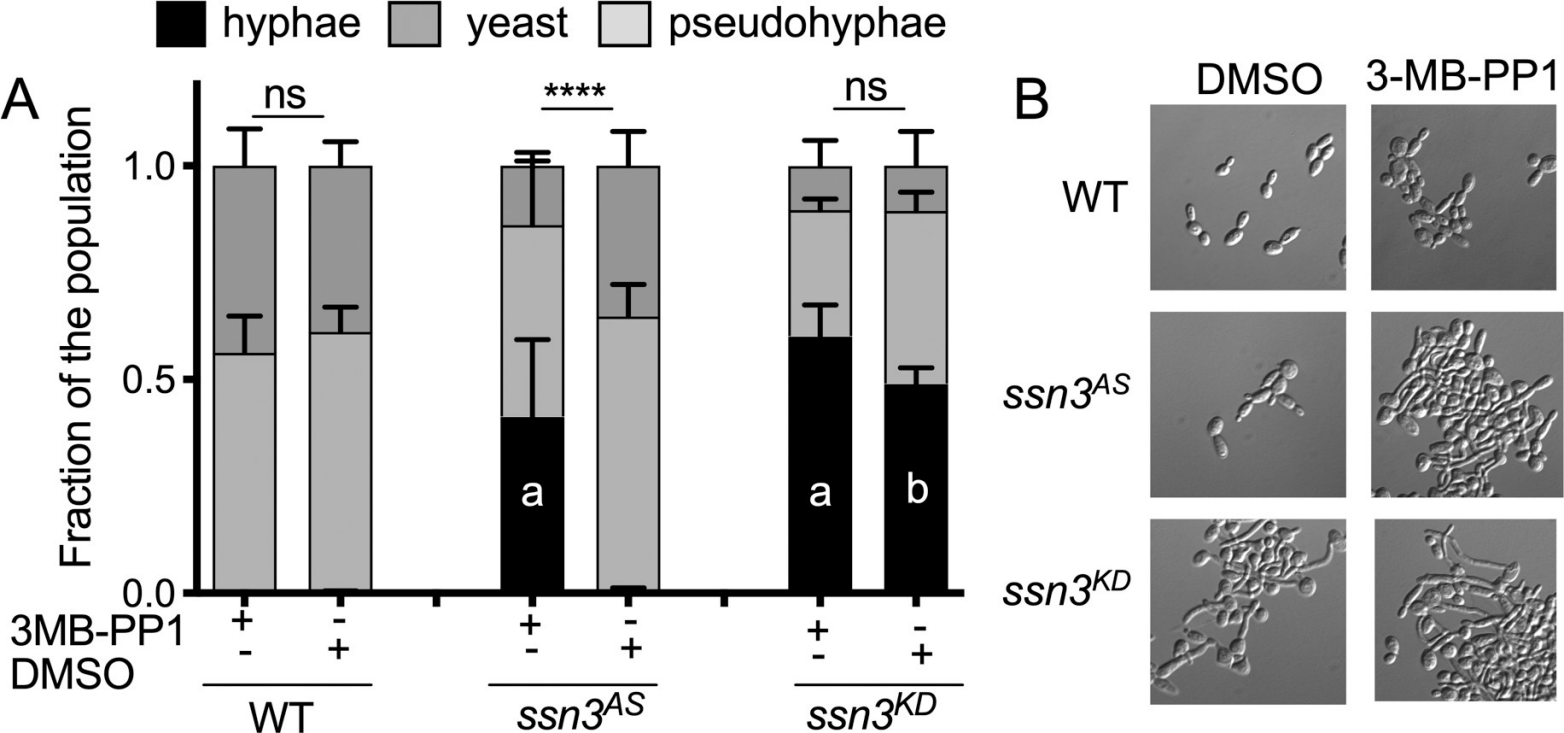
3-MB-PP1 stimulates hyphal growth in a strain bearing analog-sensitive alleles of *SSN3*. **A.** Morphology of SC5314 (WT), *ssn3*^*AS*^ and *ssn3*^*KD*^ strains was assessed after growth in YNBAG supplemented with either 5 μM 3-MB-PP1 or vehicle (DMSO) for 3h at 30°C. Quantification of yeast, pseudohyphae and hyphae in cultures was performed by microscopic analysis of blinded samples. Results from ANOVA with multiple comparisons for the percent of cells as hyphae is shown; ****, p<0.001, ns, not significant. Lower case letters indicate statistically significant differences in comparison to a) WT with 3MB-PP1 at p<0.001 or b) to WT with DMSO at p<0.001. **B.** Representative images of cell populations from cultures analyzed in panel A are shown.

To analyze Ssn3 kinase activity in live fungal cells, we engineered a derivative of *C*. *albicans* SC5314 in which both alleles of *SSN3* had been replaced by *ssn3*^*AS*^. For comparison, we also constructed a strain in which each *SSN3* allele was replaced with *ssn3-D325A*, which encodes a non-functional, or kinase dead, Ssn3 variant [22] due to mutation of a key aspartate in the catalytic site. This kinase-dead variant is referred to here as *ssn3*^*KD*^. We have previously shown that, under certain growth conditions, *ssn3Δ/Δ* mutants form hyperwrinkled colonies compared to the SC5314 wild type (WT) [13], a phenotype associated with increased hyphal growth. Thus, we asked if the Ssn3^AS^-expressing strain had a hyperfilamentous phenotype specifically in the presence of 3-MB-PP1, but not in its absence. To quantify hypha formation differences in WT, *ssn3*^*AS*^, and *ssn3*^*KD*^ strains, we grew cells in YNB containing 11 mM glucose and amino acids (YNBAG) and N-acetyl-glucosamine (GlcNAc). Both amino acids and GlcNAc are inducers of hyphal growth, but cultures were incubated at 30°C, a temperature lower than that generally used to induce robust hyphal growth. All cultures received either 3-MB-PP1 or the vehicle DMSO. The morphology of the WT was almost entirely yeast and pseudohyphae, and the relative fractions of these morphologies were unaffected by 5 μM 3-MB-PP1 (**Fig 2A and 2B**).While the morphological profiles for *ssn3*^*AS*^ cells were similar to the WT in DMSO control cultures, the addition of 3-MB-PP1 to*ssn3*^*AS*^ cultures caused a significant increase in the number of hyphae (**Fig 2A and 2B**).The increase in the fraction of cells in the hyphal morphology in 3-MB-PP1 treated *ssn3*^*AS*^ was concomitant with a significant decrease in the fraction of cells present as yeast. The *ssn3*^*KD*^ strain formed significantly more hyphae than the WT and *ssn3*^*AS*^ strains in control conditions, and the fraction of cells as hyphae was unaffected by the addition of 3-MB-PP1 (**Fig 2A and 2B**). Together, these data indicated that 5 μM 3-MB-PP1 inhibits Ssn3^AS^ *in vivo* and that the ATP analog has no discernible effects on the morphology of WT or *ssn3*^*KD*^ strains.

### Transcriptomic analyses reveal that inhibition of Ssn3 by 3-MB-PP1 leads to the induction of hypha-specific genes

**Fig 3:**
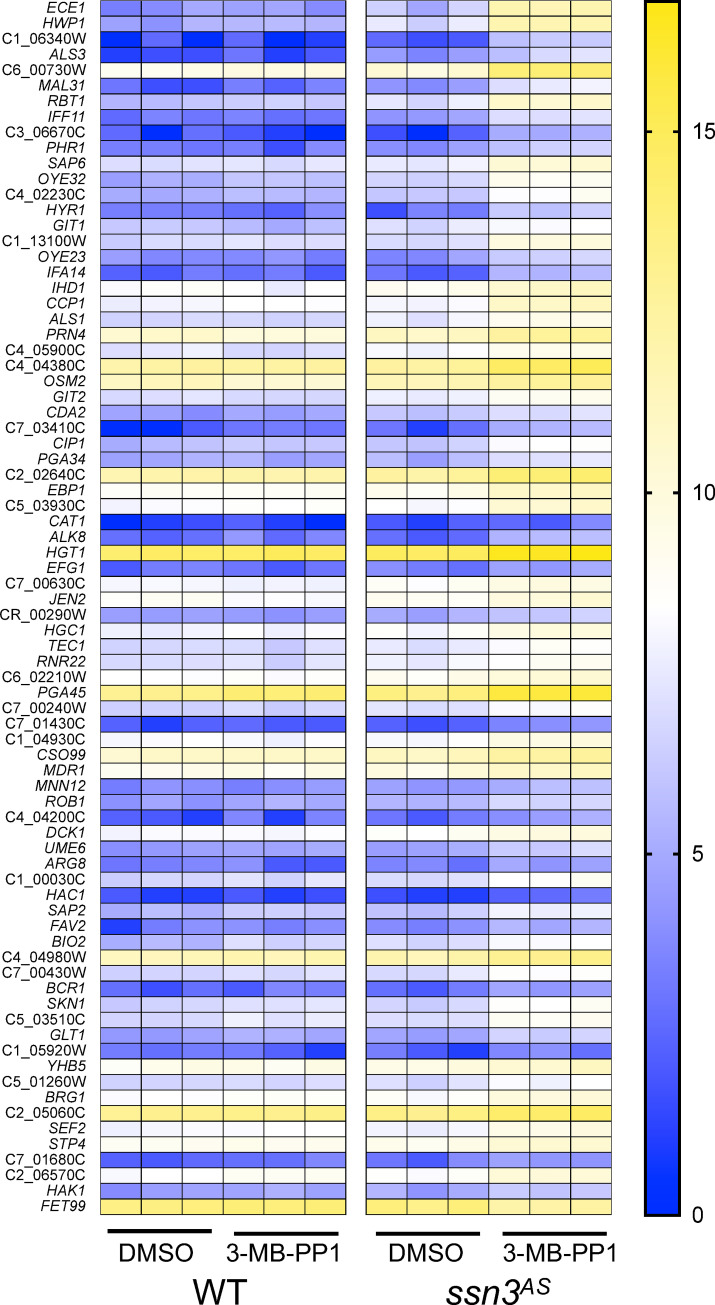
Heat map for genes induced upon inhibition of Ssn3^AS^ by 3-MB-PP1. Transcripts for seventy-three genes were 2-fold higher (p<0.05, FDR corrected) in both the comparison of Ssn3^AS^ with 3-MB-PP1 compared to WT with 3-MB-PP1 and of Ssn3^AS^ with 3-MB-PP1 compared to with the DMSO control. Genes are listed in order with the greatest magnitude difference between Ssn3^AS^ with and without 3-MB-PP1 at the top. The heat map shows three replicates per sample of Log2-transformed counts per million for expressed transcripts.

To further explore the effects of Ssn3 inhibition on the regulation of hypha formation [13] and on transcription more broadly, we examined the transcriptomes of *ssn3*^*AS*^and WT strains grown with either 3-MB-PP1 or DMSO vehicle. The WT strain was included in this experiment to assess off-target effects of 3-MB-PP1. Cells from stationary phase cultures were inoculated into the same medium used for morphology assessment, YNBAG, with either 5 μM 3-MB-PP1 or an equivalent volume of DMSO. Three replicate cultures for each strain-treatment combination were incubated for 60 min at 30°C prior to RNA harvest and sequencing as described in the Materials and Methods section. In the *ssn3*^*AS*^ strain, we found that 249 genes were significantly up regulated upon treatment with 3-MB-PP1 by 2- to 49-fold (p<0.05 FDR); transcripts for 33 genes were significantly lower by more than 2-fold with treatment (**S2 Table**). Fewer genes were affected by 3-MB-PP1 in the WT with 157 and 4 genes significantly increased and decreased, respectively; the magnitudes of the changes were also smaller (2- to 6-fold) (**S2 Table**). The greater number of transcripts at higher levels upon inhibition of Ssn3 is consistent with the Cdk8 module being a predominantly negative regulator of transcription factors [3], transcriptional re-initiation, or “pausing” of RNA polymerase II (reviewed in [23]). We found seventy-three transcripts that exhibited a significant fold increase (>2-fold) in both the comparison of the *ssn3*^*AS*^strain treated with 3-MB-PP1 to its treatment with DMSO, and the comparison of 3-MB-PP1-treated *ssn3*^*AS*^ strain to 3-MB-PP1-treated WT (**Fig 3**) with gene expression shown in log_2_-transformed counts per million). Consistent with phenotypic analysis of the effects of Ssn3 inhibition on morphology (**Fig 2**), transcripts encoding *ECE1* and *HWP1* were the two genes most highly induced by Ssn3 inhibition in the AS strain (46- and 22-fold higher, respectively) and other transcripts associated with growth in the hyphal morphology, such as *ALS1*, *ALS3*, *IHD1*, *RBT1*, *HYR1*, and *HGC1*, were also differentially-increased upon Ssn3 inhibition [15,24]. Consistent with the previous finding that Ssn3 represses induction of Mrr1 controlled genes, *MDR1* was significantly induced with Ssn3 inhibition [12]. Other transcriptional responses to the inhibition of Ssn3 activity are discussed below.

### Specific inhibition of Ssn3 affects the phosphoproteome during the induction of hyphal growth

To identify direct phosphorylation targets of Ssn3 that led to changes in phenotype and the transcriptome, we used mass spectrometry to quantitatively analyze the phosphoproteomes of the *ssn3*^*AS*^ strain compared to the WT after 3-MB-PP1 treatment. We grew *ssn3*^*AS*^ and WT cells to stationary phase, then incubated culture aliquots with 5 μM 3-MB-PP1 for five minutes to allow for drug entry into the cell. We then added concentrated, fresh, pre-warmed YNBAG medium to reproduce the conditions that induce hyphal growth in strains with low Ssn3 activity but not in the WT. Cultures were incubated for an additional 15 min at 30°C with shaking followed by rapid harvest to minimize secondary effects resulting from Ssn3 inhibition. Cells were lysed under liquid nitrogen by grinding.

To reveal the direct targets of Ssn3, we focused on phosphosites that became less abundant in the presence of 3-MB-PP1 in the *ssn3*^*AS*^ strain, but not the WT (**Tables 1** and **S3** for complete dataset). We found that 977 phosphopeptides from 552 proteins were significantly depleted in *ssn3*^*AS*^, but not WT, by more than 2-fold (P<0.05) (**Table 1**). Within this group of depleted phosphosites, 82.7% were serines, 15.8% were threonines, and 1.5% were tyrosines. These proportions are very similar to those we observed with a quantification of the whole *C*. *albicans* phosphoproteome [21], with a slight increase in the number of phosphoserines and a concomitant decrease in the number of phosphothreonines. Although promiscuous, Cdk kinases have been described as proline-directed kinases, and consistent with this, 293 phosphopeptides had phosphosites within a proline motif containing a proline in the position adjacent to C-terminal side of the phosphoresidue (**Table 1**) [25]. A much smaller number of total phosphopeptides and phosphosites in proline motifs increased upon Ssn3 inhibition (**Table 1**), which likely reflect secondary effects of Ssn3 inhibition or off-target effects on 3-MB-PP1 in the *ssn3*Δ/Δ background.

**Table 1:** Summary of phosphopeptides and phosphoproteins detected by motif. Analysis limited to those peptides to which P-values of <0.05 were assigned in the comparison of *ssn3*^*AS*^ to the wild type (WT). Down indicates phosphopeptides lower in *ssn3*^*AS*^ treated with 3-MB-PP1 compared to WT treated with 3-MB-PP1; up indicates phosphopeptides that were more abundant in the *ssn3*^*AS*^strain treated with 3-MB-PP1 compared to WT treated with 3-MB-PP1. The total refers to the number of peptides or proteins that are significantly different regardless of fold-difference. “#” indicates the detected phosphorylation on a serine or threonine, S/T, in a -1 position relative to a proline, S/T#P motif.

	Down >2-fold	Up >2-fold	Total Down	Total Up
Total phosphopeptides	977	264	2967	2522
Total phosphoproteins	552	202	1165	1057
S/T#P motif phosphopeptides	293	94	943	867
S/T#P motif phosphoproteins	218	77	505	493

**Fig 4:**
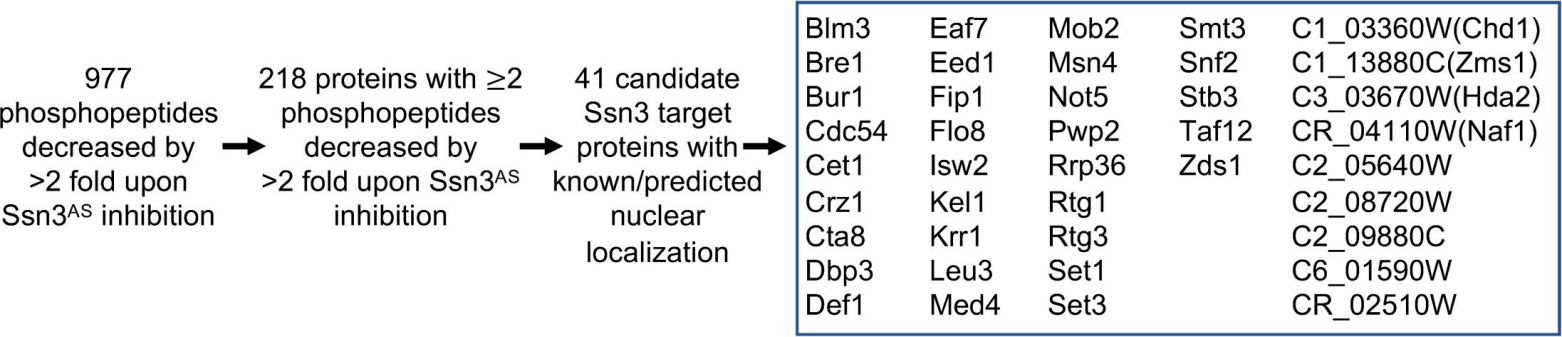
Overview of proteins for which two phosphopeptides were lower by >2 fold upon inhibition of Ssn3. Focusing on peptides that were significantly lower in Ssn3^AS^ treated with 3-MB-PP1 relative to control cultures (p<0.05 FDR-corrected), we found 977 peptides that were 2-fold lower upon 3-MB-PP1 inhibition of *ssn3*^*AS*^ relative to changes in the wild type. Two hundred and eighteen proteins had two more peptides that met these criteria, forty one of which were predicted to have nuclear localization. Med4 is a validated *C*. *albicans* Ssn3 target. If an alias is available for an unnamed gene, it is shown in parentheses.

Of the 552 proteins with phosphosites that were reduced by Ssn3 inhibition, 218 proteins contained two or more depleted phosphopeptides) in the *ssn3*^*AS*^ strain compared to WT (a consideration taken to increase stringency). Forty one of these 218 proteins were annotated as having known or predicted nuclear localization in UniProt **(Fig 4)** [26]. Among these proteins with phosphopeptides depleted upon Ssn3^AS^ inhibition was Med4, with phosphorylated S21 and S33; these Med4 sites have previously been identified as target sites for the *C*. *albicans* Ssn3 kinase [21]. We also observed depletion of specific phosphosites (T589 and S620) in Flo8, a regulator of filamentous growth in both *C*. *albicans* and *S*. *cerevisiae* [18,19,27], and both T589 and S620 were found within proline motifs (**Table 1**). The differences in Flo8 phosphopeptides were not accompanied by differences in *FLO8* mRNA levels upon3-MB-PP1 addition to the *ssn3*^*AS*^ strain (**S2 Table**).

### Flo8 is required for Ssn3-dependent hyperfilamentation and hypha-specific gene expression

**Fig 5:**
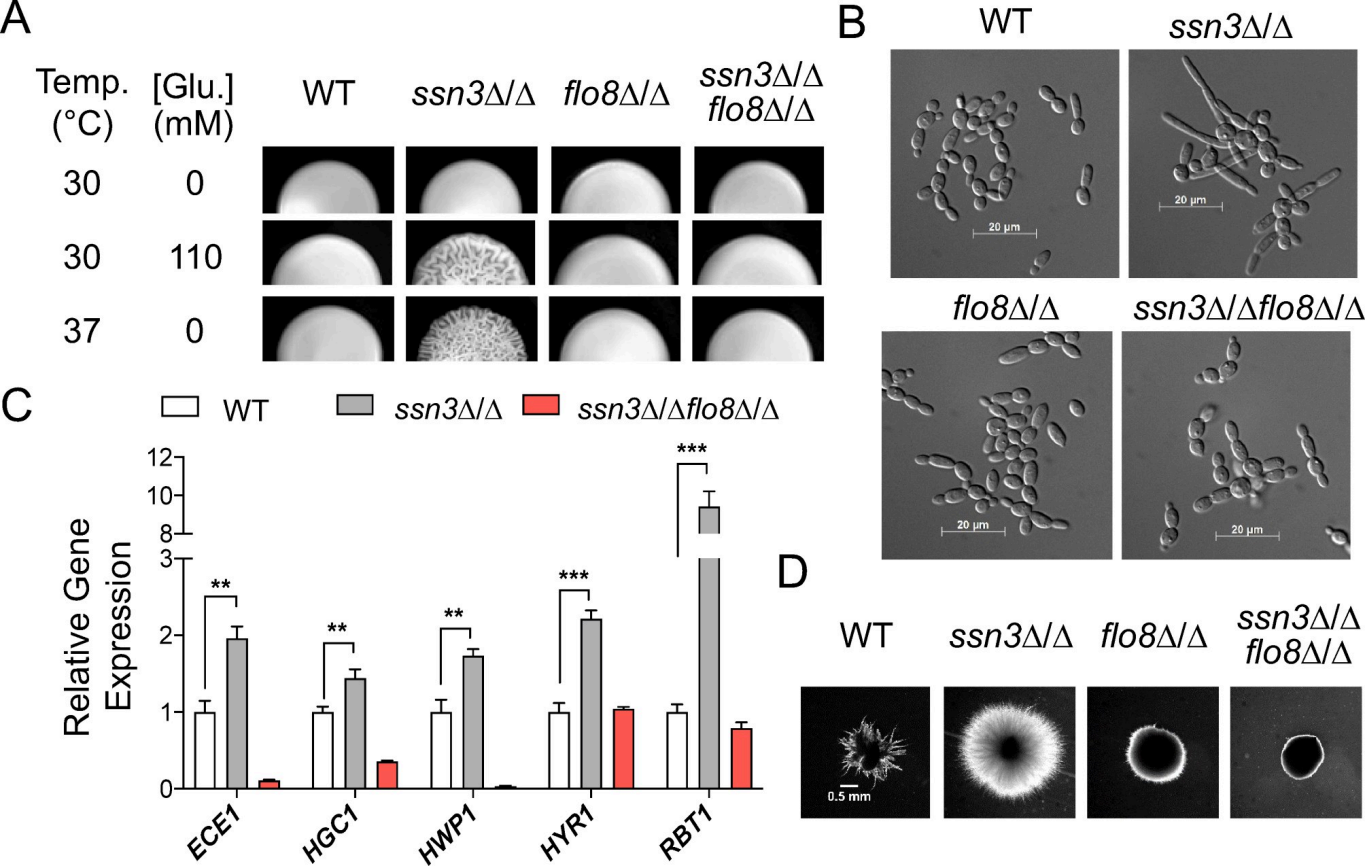
Ssn3 repression of filamentation is *FLO8* dependent. A. Colony morphology of SC5314 wild type (WT), *ssn3*Δ/Δ, *flo8*Δ/Δ, and *ssn3*Δ/Δ*flo8*Δ/Δ strains grown on YNBA agar medium alone or supplemented with 110 mM glucose at 30°C or on YNBA at 37°C. B. Cell morphology of strains tested in (A) after growth in YNBN_2.5_AG (11 mM glucose) at 30°C for 3 hours. C. NanoString analysis of indicated hypha-associated genes in WT, *ssn3*Δ/Δ, and *ssn3*Δ/Δ*flo8*Δ/Δ. RNA was extracted from cells grown as indicated for (B) but for 75 minutes. Expression levels was represented by mean and standard deviation after normalization to *TEF1* and *ACT1* reference transcripts. Expression of each gene was normalized to levels for the WT. Significant differences from WT are indicated as **, p<0.01 and ***,p<0.001. D. Embedded colonies of the same set of strains shown in (A) grown in YPS agar at 25°C.

**Fig 6:**
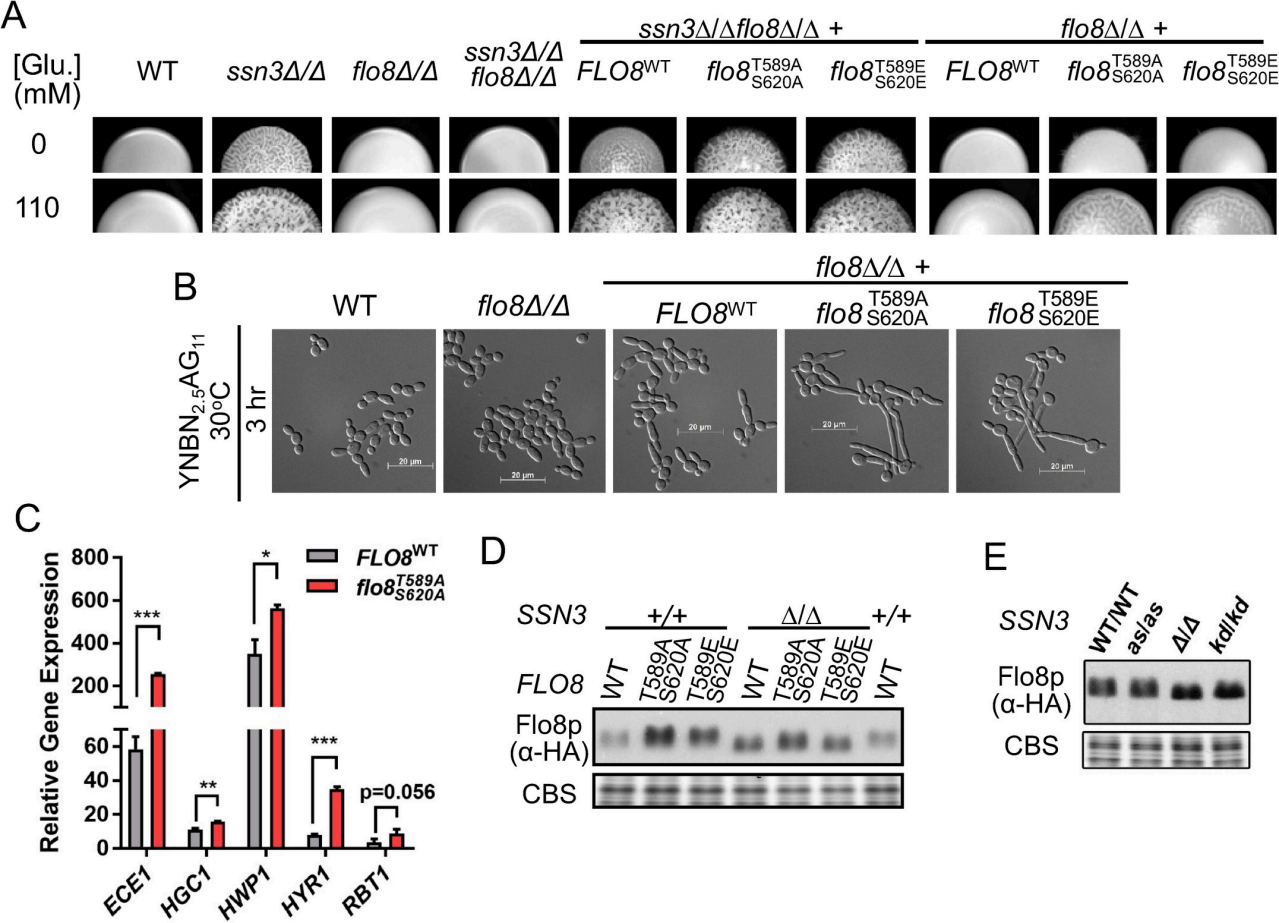
Alteration of Flo8 phosphosite residues influences Flo8 function. A.Wild type (WT), *ssn3*Δ/Δ, *flo8*Δ/Δ and *ssn3*Δ/Δ*flo8*Δ/Δ strains were imaged after growth as colonies on YNBA or YNBA+110 mM glucose at 37°C. In addition, *ssn3*Δ/Δ*flo8*Δ/Δ or *flo8*Δ/Δ were complemented with constructs that encoded Flo8^WT^, Flo8^T589A/S620A^ or Flo8^T589E/S620E^ with C-terminal 3XHA tags. B. Cell morphology of WT, *flo8Δ/Δ*, and *flo8Δ/Δ* expressing 3XHA-tagged Flo8^WT^, Flo8^T589A,S620A^ or Flo8^T589E,S620E^ after growth in YNBN_2.5_AG(11 mM glucose) at 30°C for 3h. C. Expression of hypha-associated transcripts in *flo8*Δ/Δ expressing either 3XHA-tagged Flo8^WT^ or Flo8^T589A/S620A^ after growth in YNBNAG for ~2h^.^ Data are normalized levels of the corresponding transcript in *flo8Δ/Δ*. Data show the mean and standard deviation from measurement on triplicate RNA samples. P-values, determined by t-test, indicated by *, p<0.05; **, p<0.01; ***, p<0.001; or the specific value was indicated. D. Immunoblotting analysis on 10% SDS-PAGE with an α-HA antibody shows levels and mobility of 3X-HA derivatives of Flo8, Flo8^T589A/S620A^ or Flo8^T589E/S620E^ in either WT or *ssn3*Δ/Δ. CBS indicates levels of Coomassie Blue stained total protein in a different region of the blot and is used as a loading control. E. Immunoblot of Flo8-HA levels and mobility on 10% SDS-PAGE in strains with full Ssn3 activity, WT, *ssn3*^*AS*^, *ssn3*Δ/Δ (Δ/Δ) or *ssn3*^*KD*^ (which expresses the kinase dead variant; *kd/kd*). CBS indicates levels of Coomassie Blue stained total protein in the region of the target protein, and is used as a loading control.

To determine whether there was a genetic interaction between *SSN3* and *FLO8* that accompanied the phosphoproteomic data above, we investigated the phenotype of single and double null mutants of these two genes. We previously reported that an *ssn3* null mutant forms wrinkled colonies in the presence of a metabolic inhibitor, pyocyanin, while the WT does not [13]. While both the *ssn3* null strain and the WT formed smooth colonies on YNBA agar at 30°C (which is the same as YNBAG used above but without GlcNAc), only the *ssn3Δ/Δ* strain formed wrinkled colonies at 37°C. Similarly, the *ssn3*Δ/Δ strain, but not the WT, formed wrinkled colonies on solid YNBA medium with 110 mM added glucose (**Fig 5A**). This wrinkled colony phenotype of the *ssn3*Δ/Δ mutant under the above conditions was abolished upon deletion of *FLO8* (*ssn3*Δ/Δ*flo8*Δ/Δ) (**Fig 5A**). The *flo8*Δ/Δ mutant formed smooth colonies, like the WT, under all conditions. In liquid medium, we observed a similar epistatic relationship between *FLO8* and *SSN3*. Only the *ssn3*Δ/Δ strain, and not the WT, formed hyphae and the hyperfilamentation phenotype in *ssn3*Δ/Δ was dependent on *FLO8* (**Fig 5B**). Expression levels of hypha-specific transcripts that were induced upon inhibition of Ssn3^AS^ (**Fig 3**) were significantly higher in the *ssn3*Δ/Δ background compared to the WT, but not in the ssn3Δ/Δ*flo8*Δ/Δ background (**Fig 5C**). In **Fig 6**, we demonstrate the ability to complement the filamentation defects of the *flo8*Δ/Δ and *ssn3*Δ/Δ*flo8*Δ/Δ with the native *FLO8* allele, and this result is described in more detail below.

In addition to the wrinkled colony and liquid media hyperfilamentation phenotypes, the *ssn3*Δ/Δ strain was also hyperfilamentous, compared to WT, in embedded conditions. As observed with the other hyperfilamentation phenotypes, deletion of *FLO8*in the *ssn3*Δ/Δ background was able to suppress this phenomenon (**Fig 5D**). Consistent with the results of Cao et al. [18], we observed that *FLO8* was necessary for embedded filamentation (**Fig 5D**). To determine whether *FLO8* is also required for embedded hyphal growth in *efg1*Δ/Δ, another strain that is hyperfilamentous under embedded conditions [28,29], we generated a *flo8* and *efg1* null double mutant. We found that *FLO8* disruption suppressed embedded filamentation in the *efg1Δ/Δ* background (**S1A Fig**). While Efg1 is a negative regulator of embedded hyphal growth, it is well-known for its positive regulation of hypha formation under other conditions. The *efg1*Δ/Δ mutant and *ssn3*Δ/Δ*efg1*Δ/Δ mutant strains were not filamentous in colonies grown on agar medium under filament-inducing conditions at 37°C, and the hyperfilamentous phenotype of the *ssn3*Δ/Δ mutant upon incubation under mild hypha-inducing conditions was not observed in the *ssn3*Δ/Δ*efg1*Δ/Δ mutant (**S1B Fig**). These data indicate that both Flo8 and Efg1 contribute to the hyperfilamentation of the *ssn3*Δ/Δ mutant. While we focus on Flo8 in further studies below, it is worth noting that we identified phosphosites in Efg1 (serine 531) and Ndt80 (threonines 495, 497, and 499), another hyphal growth associated transcription factor, among those that were at lower abundance upon Ssn3 inhibition (**S3 Table**).

### Loss of putative Ssn3-phosphosites in Flo8 leads to increased filamentation

To study the effects of Ssn3 activity on Flo8 protein, we complemented the *ssn3*Δ/Δ*flo8*Δ/Δ mutant with an allele that encodes a 3XHA-Flo8. This allele restored the hyperfilamentous *ssn3*Δ/Δ phenotype to the *ssn3*Δ/Δ*flo8*Δ strain (**Fig 6A**). To further explore the effects of Flo8 phosphorylation by Ssn3 we sought to determine whether Flo8 function was altered upon mutation of candidate Ssn3 target sites identified in the phosphoproteomics analysis of the Ssn3^AS^ bearing strain. Thus, we generated an allele in which both T589 and S620 were each mutated to alanine (a phospho-incompetent residue) or glutamic acid (which also abolishes phosphorylation, but sometimes can serve as a phosphomimetic). Neither the WT or *flo8Δ/Δ* strain gave a wrinkled colony morphology on either medium (**Fig 6A**) and, as expected, complementation of the *flo8*Δ/Δ with epitope tagged Flo8 did not change colony morphology. However, complementing the *flo8Δ/Δ* strain with either *flo8*^*T589A*, *S620A*^ or *flo8*^*T589E*, *S620E*^ led to a wrinkled colony phenotype in the presence 110 mM glucose, suggesting that the phosphorylation of Flo8, potentially via Ssn3, represses Flo8 activity and that the absence of these sites releases that repression (**Fig 6A**). Consistent with the increased wrinkled colony phenotype in the *flo8Δ/Δ*strains with *flo8*^*T589A*,*S630A*^ or *flo8*^*T589E*, *S620E*^ relative to the strain with unmutated *FLO8*, both Flo8 variants also led to increased hyphal development in YNBAG (**Fig 6B**). Because both *flo8*^*T589A*,*S620A*^ and *flo8*^*T589E*,*S620E*^ alleles conferred increased filamentation phenotypes consistent with high Flo8 activity in *flo8*Δ/Δ and *ssn3*Δ/Δ*flo8*Δ/Δ strains we concluded that the Flo8^T589E,S620E^variant was not acting as a phosphomimetic, but rather both had phenotypes consistent with decreased negative regulation. To quantitatively assess the differences in activity of the morphology program attributed to the loss of putative Ssn3 phosphorylation sites, levels of hypha-associated transcripts were measured in the *flo8*Δ/Δ strains with either HA-tagged *FLO8* or *flo8*^*T589A*, *S620A*^ (**Fig 6C**). We found significantly increased expression levels of several core filamentation response genes in the strain with *flo8*^*T589A*, *S620A*^ versus that with *FLO8*.

In multiple instances, phosphorylation by Ssn3 leads to decreased levels of transcription factors [6,8,16]. Thus, we tested the hypothesis that Flo8-HA^T589A,S620A^ and Flo8-HA^T589E,S620E^ were present at higher levels than Flo8-HA. Immunoblotting with an α-HA antibody indeed showed that the two variants were present at higher relative levels compared to Flo8-HA in an *SSN3* wild-type background (**Fig 6D**). Furthermore, native Flo8-HA levels were higher in the *ssn3*Δ/Δ strain than in the *SSN3/SSN3* (+/+) strain, while there were no significant differences in levels for the variant Flo8 proteins. We observed a modest increase in mobility (suggestive of decreased phosphorylation [30,31]) when Ssn3 activity was lacking (**Fig 6D and 6E**); however, because even Flo8-HA^T589A,S620A^ showed a difference in migration between WT and *ssn3*Δ/Δ (**Fig 6D**), we speculate that there are other post-translational modifications (e.g. additional phosphorylations) that influence protein mobility and that are controlled by Ssn3. It is worth noting that one other Flo8 phosphosite was identified in the phosphoproteomics analysis, but it did not reach the significance cutoff.

### Ssn3 metabolic hyperalkalinization phenotype depends on Flo8

**Fig 7:**
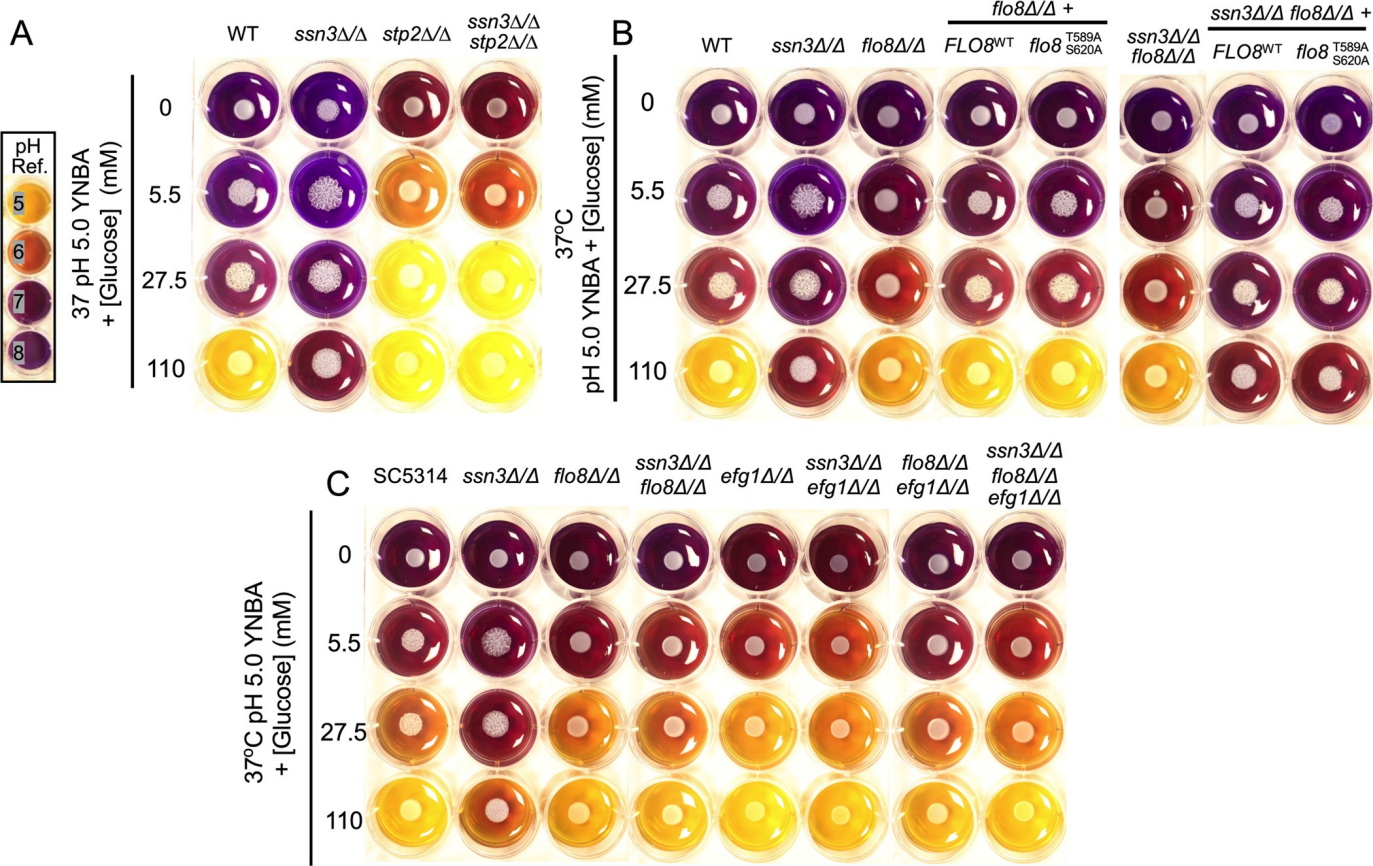
Medium alkalinization by Ssn3 is dependent on Stp2, Flo8, and Efg1. Medium pH was assessed on YNBA with increasing concentrations of glucose; bromocresol purple was added as a pH indicator. All cultures were incubated at 37°C. A. Comparison of medium pH upon growth of SC5314 (WT), *ssn3*Δ/Δ, *stp2*Δ/Δand *ssn3*Δ/Δ*stp2*Δ/Δstrains. B. WT, *ssn3*Δ/Δ, *flo8*Δ/Δ and *ssn3*Δ/Δ*flo8*Δ/Δ alone or complemented with C-terminally 3XHA-tagged Flo8^WT^ or Flo8^T589A/S620A^ were grown as described above. C. WT, *ssn3*Δ/Δ, *flo8*Δ/Δ and *ssn3*Δ/Δ*flo8*Δ/Δ were compared to *efg1*Δ/Δ, *ssn3*Δ/Δ*efg1*Δ/Δ, *flo8*Δ/Δ*efg1*Δ/Δ, and *ssn3*Δ/Δ*flo8*Δ/Δ*efg1*Δ/Δ strains.

**Fig 8:**
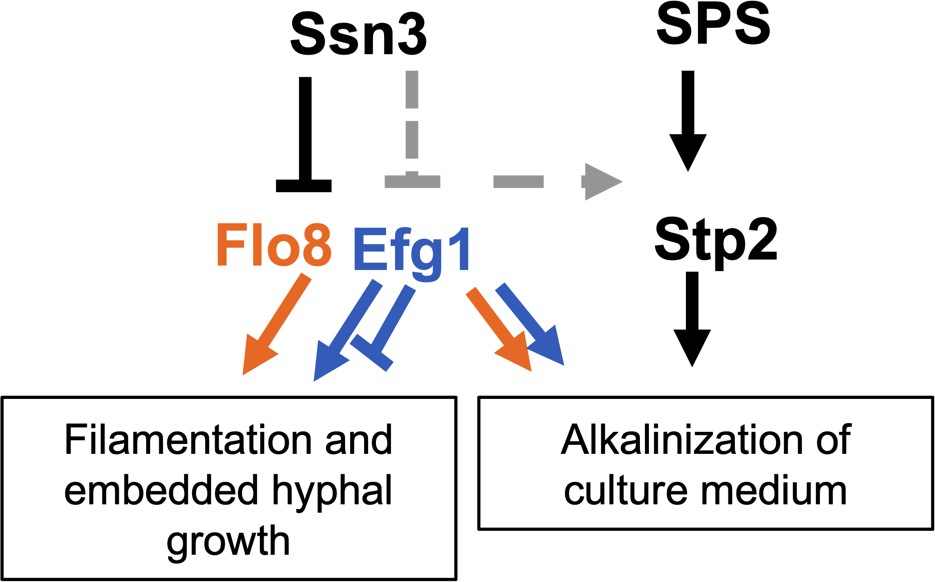
Summary of *SSN3-*centered genetic interactions revealed by this study. Hyperfilamentation of *ssn3*Δ/Δ depends on Flo8 under all conditions tested including in liquid, on agar, and in embedded conditions (orange arrow). Flo8 activity in filamentation assays is tuned at T589 and S620, which were identified as sites phosphorylated by Ssn3. Efg1 plays positive roles in the hyperfilamentation of the *ssn3*Δ/Δmutant (blue arrow) but plays a negative role in filamentation in embedded conditions (blue block). Deletion of*SSN3*also leads to hyperalkalinization of amino acid-rich media, and alkalinization is not required for filamentation. Alkalinization is reduced or no longer observed in *ssn3* mutants lacking *FLO8*, *EFG1* or *STP2*. Future studies will determine the mechanisms by which Ssn3 impacts metabolism through Flo8 and Efg1, if it directly modulates Efg1 levels (grey, dotted block arrow) and if Ssn3, Flo8 or Efg1 affect Stp2-dependent metabolism (grey dotted arrow).

Previous work has shown that the *C*. *albicans ssn3*Δ/Δ mutant had increases in several metabolic pathways including glycolysis and the utilization of amino acids relative to the WT [13], and that these contribute to a strong hyperalkalinization phenotype for *ssn3*Δ/Δ. Alkalinization of cultures is due to greater accumulation of products that raise the pH (e.g. ammonia) than products that decrease culture pH, such as the fermentation product acetic acid [32]. We constructed *stp2Δ/Δ* and *ssn3*Δ/Δ*stp2*Δ/Δ strains which lack the transcription factor Stp2, a downstream component of the SPS system that enhances ammonia release by up regulating amino acid permeases and amino acid catabolic enzymes [33]. The *stp2*Δ/Δ mutant had a defect in alkalinization at 5.5 mM glucose compared to WT, and showed medium acidification, rather than alkalinization, at both 27.5 mM and 110 mM glucose, while the WT only acidified the medium when glucose was at the highest concentration tested (**Fig 7A**). The *ssn3*Δ/Δ mutant showed the previously reported hyperalkalinization phenotype, and this phenotype was suppressed in the *ssn3*Δ/Δ*stp2*Δ/Δ strain. We found that the *ssn3*Δ/Δ*stp2*Δ/Δ strain still formed wrinkled colonies, like the *ssn3*Δ/Δ strain, suggesting that Stp2 is not upstream of Flo8 and that alkalinization is not required for hyperfilamentation (**S2 Fig**). Because Flo8 was required for hyperfilamentation in the *ssn3*Δ/Δ strain, we assessed the phenotype of the*flo8*Δ/Δ and *ssn3*Δ/Δ*flo8*Δ/Δ strains in the alkalinization assay (**Fig 7B**). While the *flo8*Δ/Δstrain did not differ from the WT in its effects on medium pH, the *ssn3*Δ/Δ*flo8*Δ/Δ cultures had lower medium pH values than the *ssn3*Δ/Δ strain, and the hyperalkalinization phenotype was restored by complementation of *FLO8* (**Fig 7A**). Unlike for filamentation, complementation of the *flo8*Δ/Δ mutant with *flo8*^*T589A*,*S620A*^ did not increase medium alkalinization relative to complementation with the native allele suggesting that Ssn3-Flo8 effects on metabolism depends on additional phosphorylation sites of Flo8 (**Fig 6D**) or that Flo8 effects on metabolism may require other factors directly or indirectly regulated by Ssn3.The *flo8*^*T589A*,*S620A*^ allele also was indistinguishable from the native *FLO8* allele in the *ssn3*Δ/Δ*flo8*Δ/Δ background. In light of our data and work by other groups that suggest complex interactions between Flo8 and Efg1 [(**S1 Fig**) and [18,34,35]] and Efg1 control of metabolism [28], we sought to determine if, like Flo8, Efg1 contributed to the hyperalkalinzation phenotype of the *ssn3*Δ/Δ mutant (**Fig 7C**). We found that *efg1*Δ/Δ and *efg1*Δ/Δ*flo8*Δ/Δ strains were similar to the WT in terms of medium alkalinization, but that the *ssn3*Δ/Δ*efg1*Δ/Δ and *ssn3*Δ/Δ*efg1*Δ/Δ*flo8*Δ/Δ strains did not show the alkalinization phenotype of the *ssn3*Δ/Δ despite robust growth. Furthermore, the phenotype of the *ssn3*Δ/Δ*efg1*Δ/Δ*flo8*Δ/Δ triple mutant was comparable to the *ssn3*Δ/Δ*efg1*Δ/Δ and *ssn3*Δ/Δ*flo8*Δ/Δdouble mutants suggesting that the effects of the *efg1*Δ/Δ and *flo8*Δ/Δ mutations were not additive. Together, these data lead us to propose that the increased alkalinization upon loss of Ssn3 activity requires both Flo8, Efg1 and Stp2 and future work will determine the specific relationships between Ssn3 and these regulators (see **Fig 8** for model). Analysis of transcripts that were differentially expressed upon inhibition of Ssn3 found a statistically significant enrichment of genes in KEGG pathways involved in amino acid metabolism, phospholipid metabolism and glycolysis (**S4 Table**) and both glycolysis and amino acid catabolism appear to be upregulated by decreased Ssn3 activity [13]. Thus, we speculate that the effects of Ssn3 on metabolism are likely multifactorial.

## Discussion

In this work, we modulated Ssn3 activity using strains with an analog-sensitive *ssn3* allele, a catalytically inactive *ssn3*^*KD*^ allele or null mutations to assess how Ssn3and its kinase activity regulate hyphal growth and metabolism. The activity of the analog-sensitive Ssn3^AS^ variant was similar to that of the native protein in both phenotype and transcriptomics analyses in the absence of 3-MB-PP1, and 3-MB-PP1 inhibition of the Ssn3^AS^ strains led to phenotypes similar to those observed in the *ssn3*Δ/Δ strain or *ssn3*Δ/Δ complemented with the kinase-dead *ssn3*^*KD*^ allele. By phosphoproteomics, we identified 552 proteins with significantly decreased phosphorylation (>2-fold, p<0.05) upon inhibition of Ssn3 during the yeast to hypha transition and they included Med4, a known Ssn3 substrate, and Flo8, a known regulator of hyphal growth [18,19]. We showed that in a variety of conditions, the loss or decrease in Ssn3 activity led to increased hypha formation and hypha-specific gene expression, and that both phenotypes were dependent on Flo8. Flo8 variants that lacked two candidate Ssn3 phosphorylation sites (T589A and S620A) caused increased filamentation and hypha-associated gene expression, supporting the model that Ssn3 is a negative regulator of Flo8. Interestingly, deletion of *JHD2*, the *S*. *cerevisiae* histone methyltransferase, and *SSN8*, which encodes the cyclin-like protein required for Ssn3 activity, results in constitutive filamentous growth that requires *S*. *cerevisiae FLO8* [10]. Our finding of 288 transcripts that meet our criteria for a statistically significant change in abundance, along with the 552 proteins with differential phosphorylation suggests that Ssn3 has numerous targets. The broad activity of Ssn3 in *C*. *albicans* mirrors the finding of Holstege and colleagues [7,36] in which microarray analyses in *S*. *cerevisiae* found ~3% of genes changed by their criteria.

In addition to Flo8, numerous of other transcriptional regulators of morphology have been described including Efg1, Cph1 (a homolog of *S*. *cerevisiae* Ste12, a known Ssn3 target), Tec1, Ndt80, and Ume6, and repressors like Tup1 and Nrg1 [37–44]. Of these, Efg1 and Ndt80 were found to have significantly depleted phosphopeptides upon Ssn3 inhibition. We focused primarily on Flo8 over Efg1 for analysis because Wartenberg *et al*. [15] recovered filamentation in a macrophage-evolved strain with *ssn3*^*R352N*^ in an *efg1/cph1* null strain background, suggesting that regulators other Efg1 and Cph1were active upon changes in Ssn3 activity. Further, we showed that in embedded growth assays, the hyperfilamenation phenotype in both the *ssn3* and *efg1* null backgrounds required Flo8, underscoring the importance of Flo8 in Efg1-independent filamentation. However, our studies indicate roles for Efg1, perhaps in conjunction with Flo8, in other filamentation phenotypes (**S1B Fig**) and in effects on metabolism (**Fig 7C**). Identification and functional characterization of Ssn3-dependent and Ssn3-independent phosphorylation sites in both Flo8 and Efg1 merits further investigation.

As research on *C*. *albicans* regulation continues, it will be interesting to determine how changes in Ssn3 activity alter the sensitivity of *C*. *albicans* to different hypha-inducing stimuli that induce activity of known hyphal growth regulators. Flo8 has been implicated in the transcriptional regulation of true hyphal growth in *C*. *albicans*, and Flo8 with its binding partner Mss11, has been described as directly binding to the Hyphal Control Region in the promoter of *HWP1*, one of the most strongly differentially abundant transcripts in our transcriptomic data. Flo8 is thought to be downstream of PKA [27,45,46]. Ssn3 may play an important role in coordinating the response of multiple regulators during the induction and repression of filamentation. A recent study identified Ssn3- dependent phosphorylation of the transcription factor Ume6 as involved in its degradation under conditions of hypoxia and atmospheric CO_2_, indicating another way in which Ssn3 can impact filamentation [16]. The absence of Ume6 phosphopeptides in our phosphoproteomics dataset could be due to differences in the kinetics of Flo8 and Ume6 phosphorylation or differences in the phosphoproteomic approach used here versus the targeted approach in the previous work [16].

In addition to hyperfilamentation, wrinkled colony formation and medium alkalinization are phenotypes associated with *ssn3* deletion. As for hyperfilamentous growth, deletion of *FLO8* and *EFG1* suppressed the hyperalkalinization phenotype of the *ssn3Δ/Δ* strain. While we show that metabolic regulator Stp2 is necessary for medium alkalinization by the *ssn3*Δ/Δ mutant, we do not yet know if Ssn3, Efg1 or Flo8 affects Stp2 or other factors involved in amino acid catabolism (**Fig 8**). The regulation of metabolism and filamentation by Ssn3 may indicate a coordinated change in cell-state required for fitness in settings where filamentation is induced. In addition to finding changes in phosphorylation of transcription factors, we found evidence for Ssn3 phosphorylation of proteins involved in the remodeling of chromatin, such as the Set3 histone deacetylase. Set3 is also involved in morphological determination, with a *set3* null mutant displaying a hyperfilamentous phenotype [47]. Additionally, it has been found that alterations in chromatin architecture participate in the interplay between Nrg1 and hypha-specific gene expression [44]. In human cells, inhibition of Cdk8 and the related kinase Cdk19 by cortistatin A identified elements of the NuA3 and NuA4 histone acetyltransferase complex amongst the depleted phosphopeptides [48]. We expect that Ssn3 phosphorylation of hyphal transcriptional regulators like Flo8 acts in concert with phosphorylation of elements of the chromatin remodeling machinery, to promote the formation of repressive chromatin structures at hypha-associated promoters.

Many of the phosphoproteins impacted by our inhibition of Ssn3 are not canonically nuclear, suggesting that Cdk8 may have a cytosolic role. This is in agreement with the work of Chen and Noble which identified a role for Ssn3 in the cytosolic phosphorylation of Sef1 [22]. As we did not observe Sef1 phosphopeptides, a documented Ssn3 phospho-target, we speculate that this is due to the fact that our experiments were performed in an iron-replete medium which suppresses this phosphorylation event [22]. Several other pathways were identified as being influenced by Ssn3 in the proteomics studies including MAP kinase pathways, which parallels human Cdk8 connection to MAPK pathways [49]. Among the MAP kinase pathway regulators and effectors identified in our data, the Hog1 MAP kinase pathway stood out (**S3 Table**); within the list of depleted phosphopeptides in the *ssn3*^*AS*^ strain were Ssk2 and Pbs2, the MAPKKK and MAPKK, respectively, of the Hog1 pathway, but Hog1 itself. We also found differences in ribosomal biogenesis and protein synthesis. The dataset contains many proteins with evidence for increased and decreased phosphorylation of targets that may impact morphogenesis-related changes to the phosphoproteome. The largest decreases in phosphosites were found in Pma1, Sep7, and the largest increase was in Yvc1, a putative vacuolar cation channel. Numerous proteins have more than one phosphosite in this dataset and with some that are highly phosphorylated in particular domains.

Unlike some other components of Mediator, which have pleiotropic effects on transcription, the role of the Cdk8 module seems to be specific to certain developmental and nutrient regulated pathways across eukarya [23]. This more specialized role has made human Cdk8 a potential drug target for several diseases. For example, inhibitors of human Cdk8 have been extensively explored as potential cancer therapies [50]. As cancer therapeutics are often administered in the context of immunosuppression, it is important to understand the impact of these compounds on *C*. *albicans*, since inhibition of Ssn3 could potentiate or attenuate the virulence of this opportunistic pathogen.

## Materials and methods

### Media and growth conditions

Strains were maintained on YPD plates, and overnight cultures for morphology and RNA seq were grown in YNB/1% glucose at 30°C in culture tubes on a roller drum. YPD plates were routinely streaked from glycerol stocks, and experiments were only conducted with overnight cultures from plates not more than 5 days old. YNB medium with 2% (w/v) casamino acids and 11 mM or 110 mM glucose (YNBAG_11_ or YNBAG_110_) with or without 5 mM N-acetyl glucosamine (GlcNAc) was adjusted to pH 5.1 (morphology and RNA seq experiments) or pH 6.0 (phosphoproteomics) with concentrated hydrochloric acid, and filter sterilized. When indicated, the pH indicator bromocresol purple was included in the medium as described in [13].Other details regarding temperature and incubation time can be found in the figure legends and in the methods sections below.

### Wrinkled colony and alkalinization assays

Overnight cultures (YPD) of each strain were washed once with water and diluted to OD_600_ 4. 8 μL of cell suspension was spotted onto the indicated medium. Images were typically taken after 2 days growth at 37°C or 3 days growth at 30°C. For alkalinization assays, 2X YNB based media was adjusted to pH 5.0 by HCl, filter sterilized and mixed with autoclaved 4% agar solution. Bromocresol purple (BCP) was added to 0.01% from a 0.1% aqueous stock into the media before 2 mL was aliquoted into each well of a 24-well plate. pH references were generated using YNB media buffered by phosphate buffer (20 mM) with known pH. Glucose concentrations 0–110 mM) were as indicated in the text and/or figure legends.

### Strain construction

All strains used in this study are listed in **S1 Table**. Primers and plasmid sequences are available upon request. Strains expressing Ssn3 that were analog-sensitive (AS) or kinase-dead (KD) variants were generated by transformation of SC5314 with a DNA fragment containing *ssn3*^*F257G*^ (*ssn3*^*AS*^) or *ssn3*^*D325A*^ (*ssn3*^*KD*^) adjacent to the SAT-FLP cassette directed to the *SSN3* locus. These constructs were transformed alongside the *Candida*-optimized CRISPR/Cas9 machinery [51] and a guide sequence targeting the nuclease to the *SSN3* open reading frame. Transformants were selected on YPD with 200 μg neourseothricin (GoldBio) and resistant colonies were regrown on the same medium, then maintained on YPD. Amplification using primers that spanned the *SSN3* locus from DNA isolated from these heterozygotes revealed the *SAT1* marker was excised during outgrowth on YPD. Heterozygous strains underwent a second transformation with the same construct using the methods described above to generate mutant homozygotes. Transformants after the second round of transformation were confirmed by PCR amplification of a ~970 base pair internal region of *SSN3* covering the region encoding residues 257 and 325 using primers Ssn3 Internal FWD and Ssn3 Internal Rev, and Sanger sequencing with the reverse primer. As in the first round, a number of these transformants had excised the resistance marker as observed by PCR. Generation of other knockout mutants was carried out using a previously described transient CRISPR-Cas9 system using a SAT-flipper selection marker [52]. To generate double mutants, the *SAT1* cassette was recycled by inducing FLP recombinase expression in YP maltose (1% yeast extract, 2% peptone, 2% maltose) for 24 hours. Generation of Flo8-HA-tagged strains was similarly accomplished through a repair construct in which the desired allele was fused to the SAT-FLP marker. For *stp2*Δ/Δ, in light of a known aneuploidy, there were three copies of *STP2* in the genome and all three were deleted [53,54].

### Mediator purification and in vitro kinase assays

Ssn8-tagged Mediator containing various *SSN3*alleles was purified and used for *in vitro* kinase assays with a GST-CTD substrate as previously described [21,55]. Amounts of the kinase were normalized by the signal on the FLAG tag.

### Analysis of C. albicans morphology

Morphological assessment was conducted in YNBNAG (11 mM glucose) at 30°C. For the analysis of the effects of 3-MB-PP1 (the ATP analog used to inhibit analog-sensitive kinases), cells grown overnight at 30°C in YNB with 1% (w/v) glucose were pelleted by centrifugation and resuspended in 5 mL YNBNAG pH 5.1 containing 5 μM 3-MB-PP1 (EMD MILLIPORE) or DMSO as a vehicle control. The cells were then incubated at 30°C for 3h in culture tubes on a roller drum, fixed in formaldehyde, and morphology quantified from image captured using differential interference contrast microscopy. Cells were considered to be true hyphae if germ tubes had parallel sides and no invagination at the junction of the filament and the mother blastospore. A minimum of 175 cells per replicate were counted, and data presented represent three independent biological replicates conducted on three different days. Cultures were inoculated to an initial density of 1x10^7^ cells per mL.

### Analysis of the C. albicans transcriptome upon Ssn3 inhibition using RNA seq

Cells were grown as described above for morphological assessment, but the incubation time was reduced to decrease indirect effects of Ssn3 inhibition. Specifically, the time following drug exposure was reduced from three hours to one hour. The 5 mL cultures were collected one hour after 3-MB-PP1 addition, pelleted by centrifugation, snap frozen in liquid nitrogen. RNA was isolated from frozen cell pellets with the MasterPure Yeast RNA Purification Kit (Epicentre MPY03100) as described in [56]. For RNA sequencing, 500 ng of total RNA was input into the Kapa mRNA HyperPrep kit (Kapa Biosystems, Wilmington, MA) and processed according to the manufacturer’s instructions. All 24 samples were multiplexed together into a single High Output 2x75bp run on a NextSeq500 instrument (Illumina, San Diego, CA). Raw reads were mapped to the *C*. *albicans* genome SC5314 (version A21-s02-m09-r04, candidagenome.org), and normalized using EdgeR. KEGG enrichment analysis was carried out using KOBAS 2.0 [57]. Data were presented after normalization by geometric mean of positive controls and geometric mean of *TEF1* and *ACT1* reads. Gene expression in a wild-type strain or a *flo8* mutant was set to ‘1’ as mentioned in the figure legends. The accession number for the data is GSE171859.

### Ssn3 phosphoproteomic experimental design

300 mL YNBNAG (11 mM glucose, pH 6.0) cultures of SC5314 and *ssn3*^*AS*^ were inoculated at a density of OD_600_ of 0.01 and grown to OD_600_ of 4.5 in flasks with shaking at 30°C, then incubated for a subsequent 4.5 hours to ensure cells had entered stationary phase. Stationary phase cells were incubated in the presence of 5 μM 3-MB-PP1 for five minutes to enable drug entry into the cell. The cells were then concentrated and added to fresh, pre-warmed medium containing either drug or vehicle and incubated for 15 minutes at 30°C with shaking. Either DMSO vehicle or 5 μM 3-MB-PP1 was then added, after which cultures were incubated with shaking for five minutes at 30°C. Then, 1.2L of prewarmed fresh YNBNAG (pH 6.0) medium was added as a 1.25X concentrate, and cells were incubated for an additional 15 minutes. The cells were then harvested by centrifugation and cell lysis by grinding under liquid nitrogen. All growth and incubation steps were conducted at 30°C, and the data represent the average of three independent replicates conducted on three separate days.

### Phosphoproteomic analysis

Yeast powder was lysed in ice-cold lysis buffer ((8 M urea, 25 mM Tris-HCl pH 8.6, 150 mM NaCl, phosphatase inhibitors (2.5 mM beta-glycerophosphate, 1 mM sodium fluoride, 1 mM sodium orthovanadate, 1 mM sodium molybdate) and protease inhibitors (1 mini-Complete EDTA-free tablet per 10 ml lysis buffer; Roche Life Sciences)) and sonicated three times for 15 sec each with intermittent cooling on ice. Lysates were centrifuged at 15,000 x *g* for 30 minutes at 4°C. Supernatants were transferred to a new tube and the protein concentration was determined using a BCA assay (Pierce-ThermoFisher Scientific). For reduction, DTT was added to the lysates to a final concentration of 5 mM and incubated for 30 min at 55°C. Afterwards, lysates were cooled to room temperate and alkylated with 15 mM iodoacetamide at room temperature for 45 min. The alkylation was then quenched by the addition of an additional 5 mM DTT. After 6-fold dilution with 25 mM Tris-HCl pH 8, the samples were digested overnight at 37°C with 1:100 (w/w) trypsin. The next day, the digest was stopped by the addition of 0.25% TFA (final v/v), centrifuged at 3500 x *g* for 30 minutes at room temperature to pellet precipitated lipids, and peptides were desalted on a 500 mg (sorbent weight) SPE C_18_ cartridge (Grace-Davidson). Peptides were lyophilized and stored at -80°C until needed for future use.

### Phosphopeptide enrichment

Phosphopeptide purification was performed as previously described [58]. Briefly, peptides were resuspended in 1.5 M lactic acid in 50% ACN (“binding solution”). Titanium dioxide microspheres were added and vortexed by affixing to the top of a vortex mixer on the highest speed setting at room temperature for 1 hour. Afterwards, microspheres were washed twice with binding solution and three times with 50% ACN / 0.1% TFA. Peptides were eluted twice with 50 mM KH_2_PO_4_ (adjusted to pH 10 with ammonium hydroxide). Peptide eluates were combined, quenched with50% ACN / 5% formic acid, dried and desalted on a μHLB OASIS C_18_ desalting plate (Waters). Phosphopeptide enrichment was repeated once.

### TMT-labeling

Phosphopeptides were resuspended in 133 mM HEPES (Sigma) pH 8.5 and 20% acetonitrile (ACN) (Burdick & Jackson). Peptides were transferred to dried, individual TMT reagent (ThermoFisher Scientific), and vortexed to mix reagent and peptides. After 1 h at room temperature, each reaction was quenched with 3 μl of 500 mM ammonium bicarbonate solution for 10 minutes, mixed, diluted 3-fold with 0.1% TFA in water, and desalted using C_18_ solid phase extraction cartridges (ThermoFisher Scientific). The desalted multiplexes were dried by vacuum centrifugation.

### Pentafluorophenyl-based Reversed Phase HPLC

Offline PFP-based reversed phase HPLC fractionation was performed as previously described [59]. Briefly, phosphopeptides were fractionated using a Waters XSelect HSS PFP 2.5 μm 2.1 × 150mm column on an Agilent 1100 liquid chromatography system, buffer A was 3% acetonitrile / 0.1% TFA, and buffer B was 95% acetonitrile / 0.1% TFA. Flow rate was 150 μl/min with a constant column temperature of 20°C. Phosphopeptides were fractioned using a 60-minute linear gradient from 8–45% acetonitrile and collected as 48 fractions between minutes 2 and 65. The 48 fractions were then combined into 24 total samples.

### TMT-based quantitative data analysis

TMT-labeled samples were analyzed on a Orbitrap Fusion [60] mass spectrometer (ThermoScientific) equipped with an Easy-nLC 1000 (ThermoScientific). Peptides were resuspended in 8% methanol / 1% formic acid across a column (45 cm length, 100 μm inner diameter, ReproSil, C_18_ AQ 1.8 μm 120 Å pore) pulled in-house across a 2 h gradient from 8% acetonitrile/0.0625% formic acid to 37% acetonitrile/0.0625% formic acid. The Orbitrap Fusion was operated in data-dependent, SPS-MS3 quantification mode [61,62] wherein an Orbitrap MS1 scan was taken (scan range = 350–1500 m/z, R = 120K, AGC target = 2.5e5, max ion injection time = 100ms), followed by ion trap MS2 scans on the most abundant precursors for 4 seconds (max speed mode, quadrupole isolation = 0.6 m/z, AGC target = 4e3, scan rate = rapid, max ion injection time = 60ms, minimum MS1 scan signal = 5e5 normalized units, charge states = 2, 3 and 4 included, CID collision energy = 33%) and Orbitrap MS3 scans for quantification (R = 15K, AGC target = 2e4, max ion injection time = 125ms, HCD collision energy = 48%, scan range = 120–140 m/z, synchronous precursors selected = 10). The raw data files were searched using COMET with a static mass of 229.162932 on peptide N-termini and lysines and 57.02146 Da on cysteines, and a variable mass of 15.99491 Da on methionines and 79.96633 Da on serines, threonines and tyrosine against the target-decoy version of the respective FASTA database (UniProt; www.uniprot.org) and filtered to a <1% FDR at the peptide level. Quantification of LC-MS/MS spectra was performed using software developed in house. Phosphopeptide intensities were adjusted based on total TMT reporter ion intensity in each channel and log_2_ transformed. P-values were calculated using a two tailed Student’s t-test assuming unequal variance.

## Supporting information

S1 Fig *FLO8* is required for hyperfilamentation in an *efg1*Δ/Δ mutant in embedded conditions. A. SC5314 wild type (WT) parental strain, *flo8*Δ/Δ, *flo8*Δ/Δ complemented with *FLO8*, *efg1*Δ/Δ, *efg1*Δ/Δ*flo8*Δ/Δ, *efg1*Δ/Δ*flo8*Δ/Δ+*FLO8* as embedded colonies in YPS agar 23°C, standard conditions for the assessment of embedded filamentation, or YNBA agar with 110 mM glucose at 30°C. The *efg1*Δ/Δ mutant is hyperfilamentous in embedded colony conditions and the filamentation is dependent on *FLO8*. B. While the WT and *ssn3*Δ/Δ strains show robust filamentation in colonies grown at 37°C on medium with 5 mM GlcNAc and 11 mM glucose, the *efg1*Δ/Δ and *ssn3*Δ/Δ*efg1*Δ/Δ mutants do not filament. On unbuffered medium with 110 mM glucose and 5 mM GlcNAc and upon incubation at a lower temperature (30°C), the *ssn3*Δ/Δ mutant is hyperfilamentous relative to the wild type and Efg1 is required for the hyperfilamentation phenotype in the *ssn3*Δ/Δ background.
(PDF)

S2 Fig The hyperwrinkled morphology of the *ssn3Δ/Δ* mutant does not require Stp2, under conditions that weakly stimulate wrinkled colony morphology in SC5314 (WT) [30°C YNBN_5_AG (11 mM glucose) agar, pH7]. In these experiments, the medium was buffered to pH 7 to eliminate differences in medium pH between the *stp2* mutants and their parental strains. All strains formed wrinkled colonies at 37°C.
(PDF)

S1 Table Strains used in this work. (PDF)

S2 Table RNA seq analyses to determine the effects of specific inhibition of Ssn3^AS^ by 3-MB-PP1 (drug). Sheets one and two compare the effect of drug versus vehicle in SC5314 WT and *ssn3*^*AS*^, respectively.
(XLSX)

S3 Table Phosphoproteomic analysis of the effect of 3-MB-PP1 on *ssn3*^*AS*^versus wild type. Sheet one provides an annotated summary of phosphopeptides statistically significantly altered by drug treatment of compared to drug treatment of wild type. Sheet two provides the data with each replicate of the triplicate experiment.
(XLSX)

S4 Table KEGG pathway enrichment analysis of transcripts altered in abundance in the *ssn3*^*AS*^ strain by 3-MB-PP1 treatment vs vehicle. (XLSX)
