# Supplementary material for: The Candida albicans Cdk8-dependent phosphoproteome reveals repression of hyphal growth through a Flo8-dependent pathway
Source: PLoS Genet. 2022 Jan 4;18(1):e1009622. doi: 10.1371/journal.pgen.1009622 (PMC8769334; doi:10.1371/journal.pgen.1009622)
Supplement: S1 Fig — A. SC5314 wild type (WT) parental strain, flo8Δ/Δ, flo8Δ/Δ complemented with FLO8, efg1Δ/Δ, efg1Δ/Δflo8Δ/Δ, efg1Δ/Δflo8Δ/Δ+FLO8 as embedded colonies in YPS agar 23°C, standard conditions for the assessment of embedded filamentation, or YNBA agar with 110 mM glucose at 30°C. The efg1Δ/Δ mutant is hyperfilamentous in embedded colony conditions and the filamentation is dependent on FLO8. B. While the WT and ssn3Δ/Δ strains show robust filamentation in colonies grown at 37°C on medium with 5 mM GlcNAc and 11 mM glucose, the efg1Δ/Δ and ssn3Δ/Δefg1Δ/Δ mutants do not filament. On unbuffered medium with 110 mM glucose and 5 mM GlcNAc and upon incubation at a lower temperature (30°C), the ssn3Δ/Δ mutant is hyperfilamentous relative to the wild type and Efg1 is required for the hyperfilamentation phenotype in the ssn3Δ/Δ background. (PDF) [file pgen.1009622.s001.pdf]

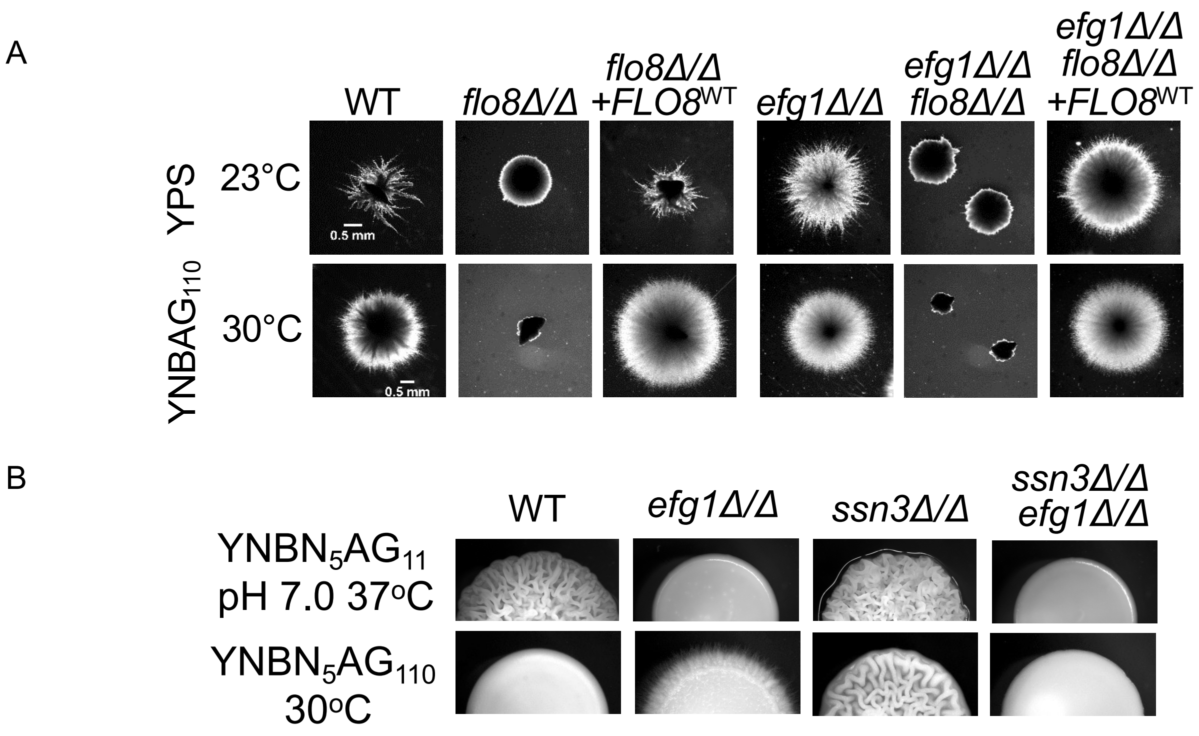

Figure S1. *FLO8* is required for hyperfilamentation in an *efg1*Δ/Δ mutant in embedded conditions. A. SC5314 wild type (WT) parental strain, *flo8*Δ/Δ, *flo8*Δ/Δ complemented with *FLO8*, *efg1*Δ/Δ, *efg1*Δ/Δ*flo8*Δ/Δ, *efg1*Δ/Δ*flo8*Δ/Δ+*FLO8* as embedded colonies in YPS agar 23˚C, standard conditions for the assessment of embedded filamentation, or YNBA agar with 110 mM glucose at 30˚C. The *efg1*Δ/Δ mutant is hyperfilamentous in embedded colony conditions and the filamentation is dependent on *FLO8*. B. While the WT and *ssn3*Δ/Δ strains show robust filamentation in colonies grown at 37˚C on buffered medium with 11 mM glucose 5 mM GlcNAc, the *efg1*Δ/Δ and *ssn3*Δ/Δ*efg1*Δ/Δ mutants do not filament. On unbuffered medium with 110 mM glucose and 5 mM GlcNAc and upon incubation at a lower temperature (30˚C), the *ssn3*Δ/Δ mutant is hyperfilamentous relative to the wild type and Efg1 is required for the hyperfilamentation phenotype in the *ssn3*Δ/Δ background.
