# Supplementary material for: The Candida albicans Cdk8-dependent phosphoproteome reveals repression of hyphal growth through a Flo8-dependent pathway
Source: PLoS Genet. 2022 Jan 4;18(1):e1009622. doi: 10.1371/journal.pgen.1009622 (PMC8769334; doi:10.1371/journal.pgen.1009622)
Supplement: S2 Fig — In these experiments, the medium was buffered to pH 7 to eliminate differences in medium pH between the stp2 mutants and their parental strains. All strains formed wrinkled colonies at 37°C. (PDF) [file pgen.1009622.s002.pdf]

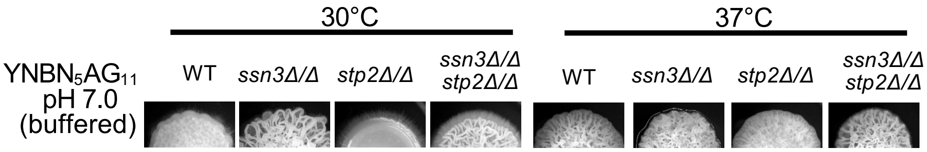

Fig. S2. The hyperwrinkled morphology of the *ssn3*Δ/Δ mutant does not require Stp2, under conditions that weakly stimulate wrinkled colony morphology in SC5314 (WT) [30°C $YNBN_5AG_{11}$ agar, pH7]. In these experiments, the medium was buffered to pH 7 to eliminate differences in medium pH between the *stp2* mutants and their parental strains. All strains formed wrinkled colonies at 37˚C.
