# Supplementary material for: The Candida albicans Cdk8-dependent phosphoproteome reveals repression of hyphal growth through a Flo8-dependent pathway
Source: PLoS Genet. 2022 Jan 4;18(1):e1009622. doi: 10.1371/journal.pgen.1009622 (PMC8769334; doi:10.1371/journal.pgen.1009622)
Supplement: S1 Table — (PDF) [file pgen.1009622.s003.pdf]

**Strain:**	**Strain number:**	**Parental strain:**	**Genotype:**
SC5314	DH35	-	Wild-type strain
ssn3^{AS}	DH2844	DH2842	*ssn3::ssn3*^{F257G}*-FRT/ssn3::ssn3*^{F257G}*-FRT*
ssn3^{KD}	DH2926	DH2842	*ssn3::ssn3*^{D325A}*-FRT/ssn3::ssn3*^{D325A}*-FRT*
ssn3Δ/Δ	DH2842	SC5314	*ssn3Δ::FRT/ssn3Δ::FRT*
flo8Δ/Δ	yLM794	SC5314	*flo8Δ::FRT/flo8Δ::FRT*
ssn3Δ/Δ flo8Δ/Δ	yLM795	DH2842	*ssn3Δ::FRT/ssn3Δ::FRT flo8Δ::FRT/flo8Δ::FRT*
FLO8-HA	yLM768	SC5314	*FLO8/FLO8::FLO8-3HA-SAT1*
ssn3Δ/Δ + FLO8-HA	yLM769	DH2842	*ssn3Δ::FRT/ssn3Δ::FRT FLO8/FLO8::FLO8-3HA-SAT1*
Ssn3^{AS} *+ FLO8-HA*	yLM770	DH2844	*ssn3::ssn3*^{F257G}*-FRT/ssn3::ssn3*^{F257G}*-FRT FLO8/FLO8::FLO8-3HA-SAT1*
ssn3^{KD} *+ FLO8-HA*	yLM771	DH2926	*ssn3::ssn3*^{D325A}*-FRT/ssn3::ssn3*^{D325A}*-FRT FLO8/FLO8::FLO8-3HA-SAT1*
flo8Δ/Δ + FLO8	yLM797	yLM794	*flo8::FLO8*^{WT}*-3HA-FRT/flo8::FLO8*^{WT}*-3HA-FRT*
flo8Δ/Δ + flo8 $^{T589A/S620A}$*-HA*	yLM798	yLM794	*flo8::flo8*$^{T589A/S620A}$*-3HA-FRT/flo8::flo8*$^{T589A/S620A}$*-3HA-FRT*
flo8Δ/Δ + flo8 $^{T589E/S620E}$*-HA*	yLM799	yLM794	*flo8::flo8*$^{T589E/S620E}$*-3HA-FRT/flo8::flo8*$^{T589E/S620E}$*-3HA-FRT*
ssn3Δ/Δ flo8Δ/Δ + FLO8-HA	yLM800	yLM795	*ssn3Δ::FRT/ssn3Δ::FRT flo8::FLO8*^{WT}*-3HA-FRT/flo8::FLO8*^{WT}*-3HA-FRT*
ssn3Δ/Δflo8Δ/Δ + flo8 $^{T589A/S620A}$*-HA*	yLM801	yLM795	*ssn3Δ::FRT/ssn3Δ::FRT flo8::flo8*$^{T589A/S620A}$*-3HA-FRT/flo8::flo8*$^{T589A/S620A}$*-3HA-FRT*
ssn3Δ/Δflo8Δ/Δ + flo8 $^{T589E/S620E}$*-HA*	yLM802	yLM795	*ssn3Δ::FRT/ssn3Δ::FRT flo8::flo8*$^{T589E/S620E}$*-3HA-FRT/flo8::flo8*$^{T589E/S620E}$*-3HA-FRT*
stp2Δ/Δ	yLM785	SC5314	*stp2Δ::NAT/stp2Δ::NAT/stp2*$_{3rd\text{-}copy}$*Δ::NAT*
ssn3Δ/Δstp2Δ/Δ	yLM786	DH2842	*ssn3Δ::FRT/ssn3Δ::FRT stp2Δ::NAT/stp2Δ::NAT/stp2*$_{3rd\text{-}copy}$*Δ::NAT*
flo8Δ/Δefg1Δ/Δ + FLO8-HA	yLM814	yLM797	*flo8::FLO8*^{WT}*-3HA-FRT/flo8::FLO8*^{WT}*-3HA-FRT efg1Δ::NAT/efg1Δ::NAT*
efg1Δ/Δ	yLM773	SC5314	*efg1Δ::NAT/efg1Δ::NAT*
flo8Δ/Δefg1Δ/Δ	yLM807	yLM794	*flo8Δ::FRT/flo8Δ::FRT efg1Δ::NAT/efg1Δ::NAT*
ssn3Δ/Δefg1Δ/Δ	yLM774	DH2842	*ssn3Δ::FRT/ssn3Δ::FRT efg1Δ::NAT/efg1Δ::NAT*
ssn3Δ/Δflo8Δ/Δefg1Δ/Δ	yLM808	yLM795	*ssn3Δ::FRT/ssn3Δ::FRT flo8Δ::FRT/flo8Δ::FRT efg1Δ::NAT/efg1Δ::NAT*
